# M. Andral on the Cicatrization of Tuberculous Excavations in the Lungs

**Published:** 1826-07

**Authors:** 


### ON THE CICATRIZATION OF TUBERCULOUS EXCAVATIONS IN THE LUNGS.*

Once developed in the pulmonary parenchyma, tubercles progressively soften down, in the great majority of cases, and make their way into the bronchia, leaving in their place an excavation of greater or less extent, which becomes larger by union with others in the neighbourhood. In general, these cavities shew no disposition to fill up or heal. The purulent secretion from them is a constant source of emaciation, and the patient ultimately sinks. Laennec has lately drawn the attention of the profession to a few exceptions from this general rule, where the excavations have healed in a manner analogous to the healing of an abscess in any external part. The truth of this fortunate event is, however, still denied by many physicians, or at all events doubted, and ranged among those phenomena which need farther proof and elucidation. On this account M. Andral considers it desirable to accumulate all the facts we can upon so interesting a point of pathology, in order that the validity of the phenomenon may be established, or its fallacy detected.

* M. Andral, jun. Revue Medicale, Decembre, 1825.

*Case* 1. A woman, about 50 years of age, died with all the symptoms of pulmonary consumption. The superior and middle lobes of the right lung presented a red induration with black spots. In the midst of this condensed portion were found some crude tubercles of small size, and five or six considerable cavities filled with pus, most probably from softened down tubercles. The left lung appeared, at first sight, to be sound; but, on minute examination, there was found, quite at the summit, a small cavity lined with a smooth, dense membrane, into which two bronchiæ opened. This cavity would hardly admit a small nut, and contained a little serous fluid. Around this cavity, the parenchymatous structure of the lung was indurated. On more accurate investigation it appeared that this cavity was only the remains of a larger one, whose parietes had coalesced, and, therefore, it was probable that had the patient lived some time longer, the whole would have been obliterated.

*Case* 2. In the body of a man, 52 years of age, the two lungs contained a great number of tubercles, crude and softened. The summit of the right lung was dark coloured, indurated, and presented a hollow or sulcus on its surface. On cutting into this part, they found, a few lines from the periphery, an irregularly roundish cavity, capable of containing a nut, communicating, by means of a short fistulous tube, with a second and smaller cavity crossed by a septum. Both of these cavities contained a reddish serum. They were surrounded for some lines in extent, with a homogeneous white substance resembling cartilage. These cavities were lined with a fine polished membrane. Three large bronchia were found running towards this indurated portion, in which they were abruptly lost or obliterated. One other bronchium, however, was found to communicate, by means of a small membranous conduit, with the larger cavity. There is every reason to believe that these were the remains of tuberculous excavations formed at some former period of the patient's life.

*Case* 3. A man, about 40 years of age, died of acute pneumonia. The greater part of the right lung was in a state of purulent infiltration. Towards the superior part of the inferior lobe of this lung, a space was found, occupied by a white fibro-cartilaginous substance, about two and a half inches in length, by two in breadth and depth. It was intimately connected on all sides with the parenchymatous structure of the lung, except at one part, where it was separated by a small oval cavity, filled with purulent matter. Into this point a large bronchial tube entered, communicating with the cavity abovementioned. There was no trace of tubercles in any other part of the lungs. We cannot but agree with M. Andral that, had this man lived some years longer, the cavity in question would have been converted into the same kind of substance with that in its immediate vicinity.

*Case* 4. In this case, where the lungs contained tubercles in various stages of advancement, M. Andral found in one of the superior lobes a production exactly similar to that described in the last case, but without any trace of cavity in it or near it. A large bronchium approached this new structure, and there became abruptly annihilated. In two or three other cases there was found a kind of line or cicatrix, as if from a wound, to which places two or more bronchia appeared to direct their course, and there became amalgamated with the adventitious tissue.

*Case* 5. A man, 24 years of age, appeared to be in the last stage of phthisis, and died two days after entering the hospital. Both lungs were found filled with tubercles, crude or softened. The summit of the right lung was hard and indented on the exterior. Incised at this point several black masses presented themselves, containing soft tubercles in their centres. Several large bronchia converged towards these, and the moment they entered into the melanose substance, their calibre was suddenly diminished or rather annihilated, and they were transformed, as it were, into ligamentous cords.

A number of other cases, more or less similar, are related, but we deem it unnecessary to adduce more examples. Enough has been brought forward here and on other occasions to render it highly probable (for the thing hardly admits of positive proof) that tuberculous excavations occasionally close up and heal. But, in admitting this, we are forced to admit the melancholy truth that, in the majority of cases, where this sanative process has been effected by Nature, it has been of little use to the unfortunate patient—in consequence of the simultaneous existence of other tubercles. The obliteration of an excavation can only be of use, where it is a single excavation, and where no other tubercles exist to go on to the same pathological condition. Such a case fortunately does sometimes occur—possibly more frequently than we imagine. We every now and then meet with patients who are spitting purulent matter, and exhibiting all the symptoms of phthisis pulmonalis. We set them down as lost cases, and give a gloomy prognosis to the friends. But, after a time, the expectoration diminishes, the other symptoms subside, and we conclude that we were mistaken in taking an affection of the mucous membrane of the lungs for a suppuration of tubercles in their substance. Is it not possible that the healing of the tuberculous excavation sometimes takes place in these cases, and that we are not so much mistaken in the nature of the disease, as in the mode of its cure?

*Case* 6. A woman died, at the age of 40, of cancer in the stomach. Several years previously, this woman was affected with all the usual symptoms of phthisis, and her physicians considered her case as hopeless. Nevertheless, the phthisical symptoms gradually declined, and Madame —— ceased, at length, to have any cough or expectoration. In the succeeding years the affection of the stomach made progress, and ultimately destroyed her life, but she evinced no symptoms of the pulmonic affection to the last.

The dissection was made by M. Bauniez, in presence of Dr. Chomel and M. Reynaud. There was cancer of the stomach, which it is unnecessary here to describe. The lungs were sound throughout almost the whole of their extent. Their summits were adherent, on both sides, to the ribs, and at these places presented a blackish colour, and a remarkable depression. In the right lung was a small induration, in which was observed a roundish body of cretaceous consistence, the parenchymatous structure of the lung being quite sound in its vicinity. Contiguous to this were two small tubercular masses. At the summit of the left lung a similar induration was found, in the midst of which was observed a small quantity of tuberculous matter, contained in a circumscribed cavity. There was, on this side, no detached tubercle. These may fairly be considered as vestiges of the patient's former pulmonary complaint.

The foregoing facts are calculated, if not to inspire hope, at least to render us cautious of giving a decidedly fatal prognosis in all cases of apparent phthisis pulmonalis. If, by percussion, auscultation, and other modes of investigation, we find that the lungs are sound, except in one particular spot, there is still a ray of hope that Nature, assisted by art, may triumph over the disease.

